# Role of Tellurite Resistance Operon in Filamentous Growth of *Yersinia pestis* in Macrophages

**DOI:** 10.1371/journal.pone.0141984

**Published:** 2015-11-04

**Authors:** Duraisamy Ponnusamy, Kenneth D. Clinkenbeard

**Affiliations:** Department of Veterinary Pathobiology, Center for Veterinary Health Sciences, Oklahoma State University, Stillwater, OK 74078, United States of America; University of Freiburg, GERMANY

## Abstract

**Background:**

*Yersinia pestis* initiates infection by parasitism of host macrophages. In response to macrophage infections, intracellular *Y*. *pestis* can assume a filamentous cellular morphology which may mediate resistance to host cell innate immune responses. We previously observed the expression of *Y*. *pestis* tellurite resistance proteins TerD and TerE from the *terZABCDE* operon during macrophage infections. Others have observed a filamentous response associated with expression of tellurite resistance operon in *Escherichia coli* exposed to tellurite. Therefore, in this study we examine the potential role of *Y*. *pestis* tellurite resistance operon in filamentous cellular morphology during macrophage infections.

**Principal Findings:**

*In vitro* treatment of *Y*. *pestis* culture with sodium tellurite (Na_2_TeO_3_) caused the bacterial cells to assume a filamentous phenotype similar to the filamentous phenotype observed during macrophage infections. A deletion mutant for genes *terZAB* abolished the filamentous morphologic response to tellurite exposure or intracellular parasitism, but without affecting tellurite resistance. However, a *terZABCDE* deletion mutant abolished both filamentous morphologic response and tellurite resistance. Complementation of the *terZABCDE* deletion mutant with *terCDE*, but not *terZAB*, partially restored tellurite resistance. When the *terZABCDE* deletion mutant was complemented with *terZAB* or *terCDE*, *Y*. *pestis* exhibited filamentous morphology during macrophage infections as well as while these complemented genes were being expressed under an *in vitro* condition. Further in *E*. *coli*, expression of *Y*. *pestis terZAB*, but not *terCDE*, conferred a filamentous phenotype.

**Conclusions:**

These findings support the role of *Y*. *pestis terZAB* mediation of the filamentous response phenotype; whereas, *terCDE* confers tellurite resistance. Although the beneficial role of filamentous morphological responses by *Y*. *pestis* during macrophage infections is yet to be fully defined, it may be a bacterial adaptive strategy to macrophage associated stresses.

## Introduction

The Gram-negative bacterium *Yersinia pestis*, the etiologic agent of plague, is maintained in nature in rodents with their associated fleas serving as vectors[1]. Following subcutaneous infection of rodents via bites of fleas infected with *Y*. *pestis*, the bacterium is phagocytized by tissue neutrophils and macrophages[2]. Although neutrophils initially kill most phagocytized *Y*. *pestis*, the bacterium is able to survive and multiply in rodent macrophages and to be disseminated to local and regional lymph nodes via migration of these macrophages [3,4,5]. Intracellular *Y*. *pestis* is able to suppress macrophage pro-inflammatory cytokine expression thereby dampening host innate immune response to the initial infection [4,6]. If the infection is not controlled by the innate immune response at this stage, then septicemic dissemination with severe disease can result. At 36 to 48 h post-infection, intracellular *Y*. *pestis* kill infected macrophages and are released initiating septicemia [4]. *Y*. *pestis* parasitism of macrophages in local and regional lymph nodes is a crucial step in plague pathogenesis [7].

Although *Y*. *pestis* is able to survive and multiply in rodent macrophages, the bacterium must overcome the harsh environment of the phagolysosome in order to sustain infection of the host. *Y*. *pestis* is readily phagocytized by macrophages during the initial phase of infection and resides in phagosomes which fuse with lysosomes to become phagolysosomes [8]. In this environment, *Y*. *pestis* experiences an acidic environment depleted of Ca^2+^, Mg^2+^, and Fe^2+^ and containing reactive oxygen species and other antimicrobial activities [9]. In response to this harsh environment, *Y*. *pestis* induces expression of multiply stress response proteins including regulatory, nutrient acquisition, detoxifying, and repair activities [10,11]. Initially, *Y*. *pestis* is contained in tightfitting phagolysosomal Yersinia containing vacuoles (YCV), but several hours post-infection (p.i.), *Y*. *pestis* mediates modification of these tight YCV to spacious YCV likely diminishing antimicrobial activities of the phagolysosomes by dilution of their antimicrobial components [8].

A novel bacterial stress response survival strategy for intracellular bacterial pathogens has recently been proposed to be morphological plasticity, and in particular, assumption of filamentous cellular morphology [12]. Previously, Janssen and Surgalla observed filamentous *Y*. *pestis* in peritoneal macrophages in experimentally infected guinea pigs, and Brubaker and colleagues noted that *Y*. *pestis* grown under Ca^2+^ deficient conditions mimicking those of the phagolysosomal environment assumed a filamentous cellular morphology [13,14]. We recently observed that approximately 7% of *Y*. *pestis* present in mouse primary or tissue culture RAW264.7 macrophages exhibited filamentous morphology lacking septa at 2.5 h p.i. [15]. This filamentous morphology also correlated with multiply genome equivalents (GE) per bacterium. By 7.5 h p.i. when *Y*. *pestis* had overcome the initial macrophage imposed stress and caused spacious expansion of YCVs, <5% of *Y*. *pestis* exhibited filamentous morphology. Those filamentous forms present at 7.5 h p.i. typically had septal divisions evident between nucleoids. By 27.5 h p.i., intracellular *Y*. *pestis* in mouse macrophages reverted to a coccobacillary cellular morphology characteristic for *Y*. *pestis*, and bacterium contained single GE.

In situations of environmental imposed stress, *Y*. *pestis* respond by induction of stress responses. One such response induced by stress associated with intracellular growth in macrophages is expression of the tellurite resistance operon proteins TerD and TerE [16]. Tellurium (Te) is a rare earth metal which forms tellurite oxyanions (TeO_3_^2-^) highly toxic to bacteria [17]. In the presence of TeO_3_^2-^, tellurite resistant bacteria possessing the *ter* operon reduce TeO_3_^2-^ to black metallic Te^0^which is deposited along the outer surface of the inner membrane or in the periplasmic space [18]. However, because of its rarity in earth environments, most bacterial species are seldom, if ever, exposed to tellurite, and the role of tellurite resistance proteins has been an enigma [17]. It has been proposed that the *ter* operon may detoxify antimicrobial compounds during oxidative stress [19]. Exposure of *E*. *coli* strain possessing tellurite resistance to tellurite caused the induction of bacterial antioxidant defense mechanism, including increased expression of superoxide dismutase, catalase, oxidoreductase and cysteine desulfurase [20,21].This proposed function is supported by the observation that expression of the *ter* operon in *Proteus mirabilis* is associated with oxidative stress as well as tellurite exposure [22]. In uropathogenic *E*. *coli*, the *ter* operon affords a survival benefit for the bacterium in macrophages [23].Similarly, in *Bacillus anthracis* mutational inactivation of the genes *yceGH*, which provide tellurite resistance for this organism, resulted in increased sensitivity to hydrogen peroxide and the antimicrobial peptide cathelicidin [24]. In addition, these mutants were attenuating for *in vitro* exposure of *B*. *anthracis* to human whole blood as well as mouse and *Caenorhabidits elegans* animal models of infection[24].

The *ter* operon also plays a role in forming bacterial filamentous cellular phenotype at least in laboratory *E*. *coli* strains[25]. Transformation of *E*. *coli* strain DH5α, which does not possess a *ter* operon, with a *ter* operon (*terZABCDEF*) from the 272 kb R478 self-transmissible plasmid of the incompatibility subgroup IncHI2 resulted in adoption of a filamentous morphology for the transformed bacterium [25]. Deletion of the *terZAB* genes from the transformants restored normal rod morphology to the bacterium. Further, it has been observed that transformation of *E*. *coli* DH5α with either *terZABCDEF* or *terZA* necessitates co-transformation of the *terXYW* genes in order to avoid lethality associated with expression of the former gene clusters. Based on these findings, we speculated that the *Y*. *pestis* filamentous morphologic response in macrophages may be mediated by the *ter* operon. To test this hypothesis, *terZAB* and *terZABCDE* deletion mutants were constructed in *Y*. *pestis* strain KIM62053.1+, and the tellurite resistance determined as well as the cellular morphology in both microbial culture and during intracellular parasitism of RAW264.7 cells. Filamentous response to tellurite exposure or intracellular parasitism was found to involve *terZAB*, whereas *terCDE* were required for tellurite resistance. The tellurite resistance operon mediation of a filamentous morphology by *Y*. *pestis* during early infection of macrophages may offer a survival benefit for the bacterium in this harsh intracellular environment.

## Results

### *Y*. *pestis ter* mutant phenotypes

**Fig 1:**
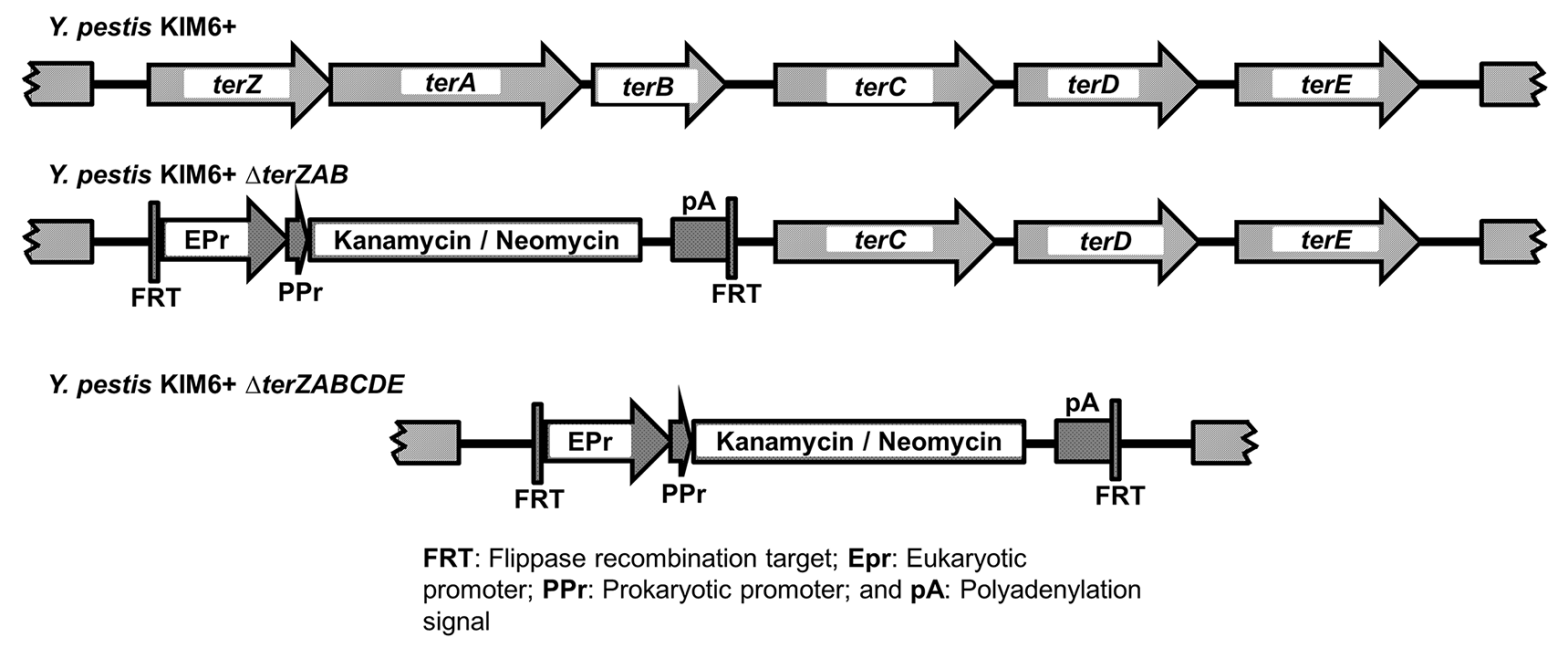
Schematic of *Y*. *pestis terZABCDE* operon and *ΔterZAB* and *ΔterZABCDE* deletion mutants.

We previously observed that TerD and TerE proteins were present in intracellular *Y*. *pestis* in RAW264.7 mouse macrophage cells at 8 h p.i., but not in extracellularly grown *Y*. *pestis* [16] and that approximately 7% of intracellular *Y*. *pestis* in RAW 264.7 cells assumed a filamentous cellular morphology[15]. Transformation of *E*. *coli* strain DH5α lacking a *ter* operon with a *ter* operon (*terZABCDEF*) from the R478 self-transmissible plasmid resulted in adoption of a filamentous morphology for the transformed bacterium [25]. To assess the role of *ter* operon in the intracellular filamentous response of *Y*. *pestis* in macrophages, partial and full-length deletion mutants for the *ter* operon were constructed and assessed for tellurite resistance, tellurite reduction phenotype, and filamentous morphologic phenotype (Fig 1). The partial-length Δ*terZAB* mutant and full-length Δ*terZABCDE* mutant exhibited similar and not statistically different growth curves to that for the parent KIM6+ in BHI broth when grown at 26°C or 37°C, although the Δ*terZABCDE* mutant had slightly lower stationary phase OD_600nm_ at 26°C than did the KIM6+ parent and the Δ*terZAB* mutant (S1A Fig). In addition, the KIM6+ parent and Δ*terZAB* mutant had slightly flocculent growth in broth culture grossly at 37°C, but the Δ*terZABCDE* mutant did not (S1B Fig).

**Fig 2:**
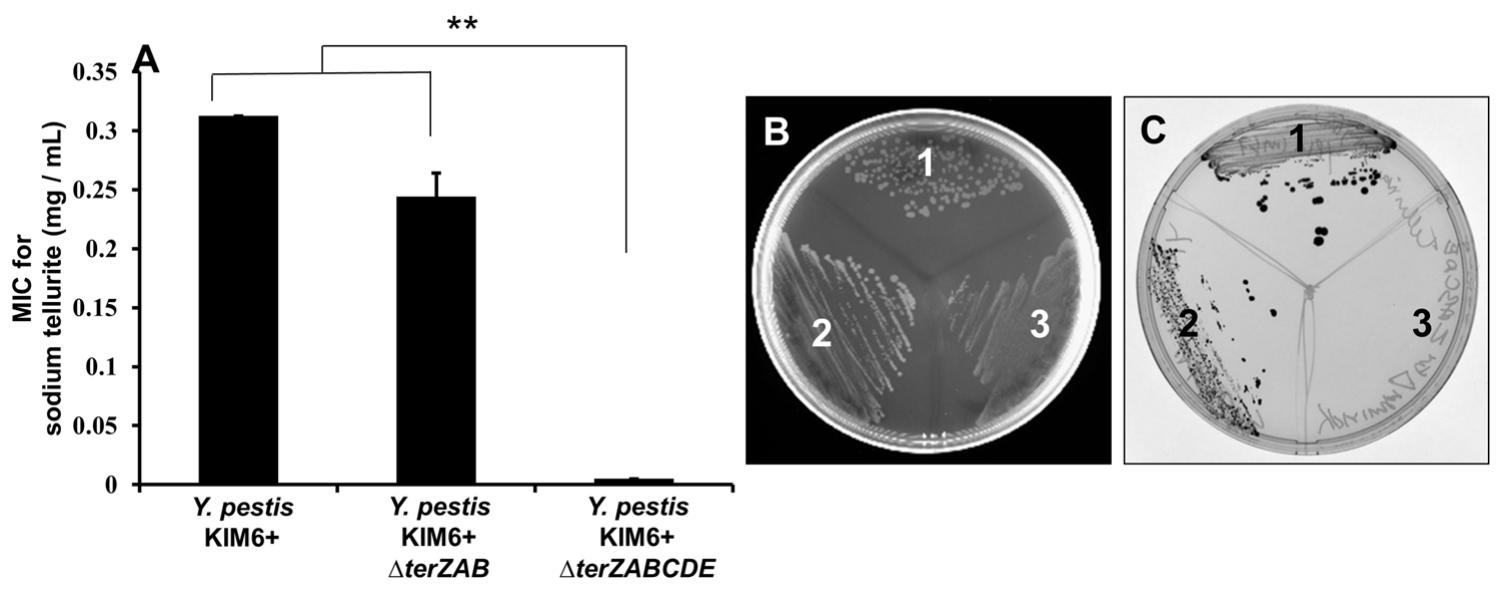
Minimal inhibitory concentrations and reductive phenotypesof*Y*. *pestis* KIM6+ and *ΔterZAB* and *ΔterZABCDE* mutants for Na_2_TeO_3_exposure. (A) *Y*. *pestis* KIM6+ and *ΔterZAB* and *ΔterZABCDE* mutants were exposed to 2-fold serial dilutions of Na_2_TeO_3_overnight at 28°C, and the MIC determined as the lowest tellurite concentration with no growth observed. Asterisk represents significant difference between the mean values (** denotes p<0.01). LB agar plates containing 0.1mg/mL of Na_2_TeO_3_(C) or equivalent volume of PBS (B) inoculated with *Y*. *pestis* KIM6+ (1) and *ΔterZAB* (2) and *ΔterZABCDE* (3) mutants were incubated at 28°C for 2 to 3 days. Black colony color was associated with tellurite reductive phenotype.

**Fig 3:**
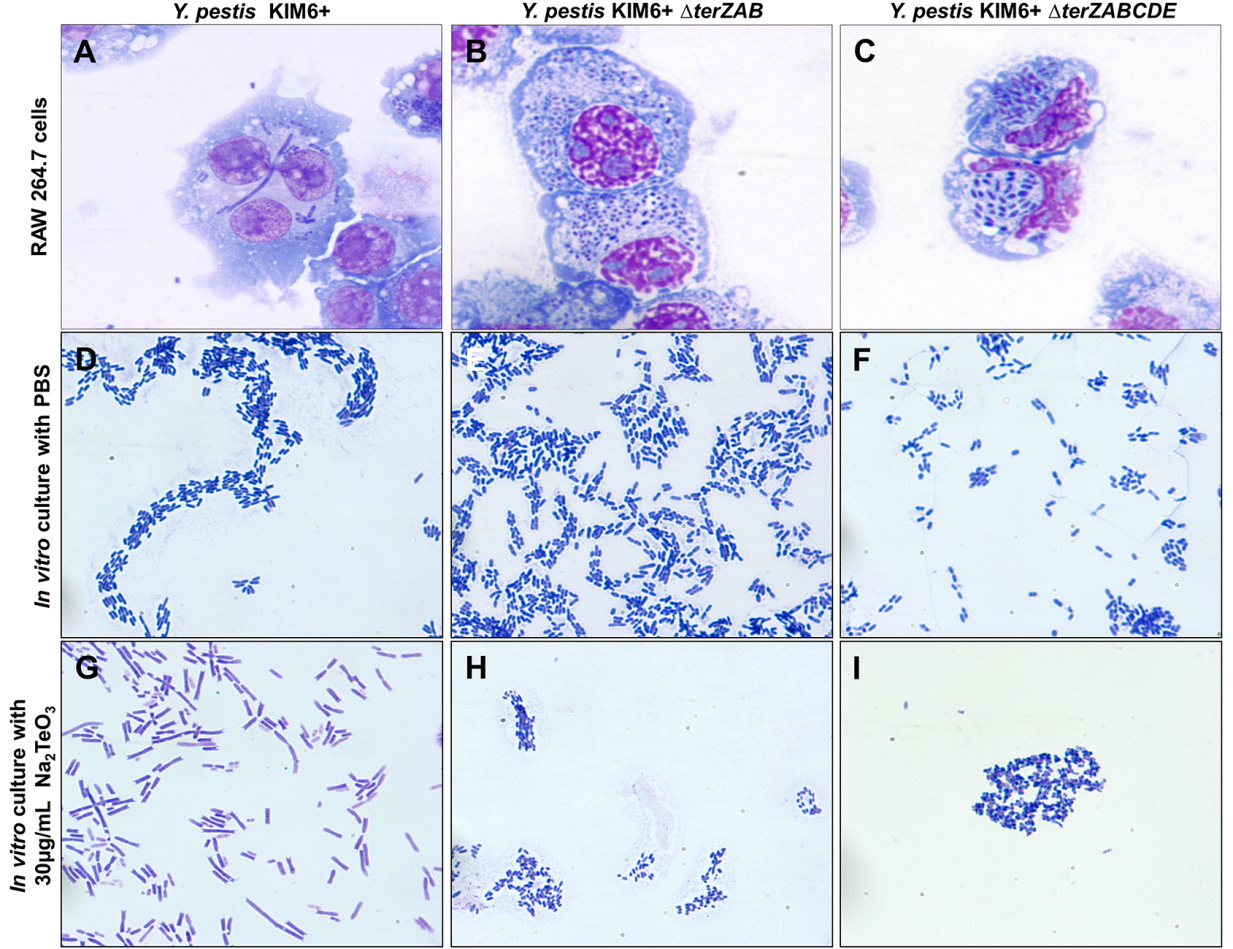
Cellular morphology of *Y*. *pestis* KIM6+ and *ΔterZAB* and *ΔterZABCDE* mutants during intracellular parasitism or in extracellular growth media. For panels A-C, *Y*. *pestis* KIM6+ (A) and *ΔterZAB* (B) and *ΔterZABCDE* (C) mutants were used to infected RAW264.7 cells. For panels D-I, *Y*. *pestis* KIM6+ (D and G) and *ΔterZAB* (E and H) and *ΔterZABCDE* (F and I) mutants cultured in RPMI-1640 media without (panels D-F) or with 0.03 mg/mL Na_2_TeO_3_ (panels G-I). Samples were collected at 2.5 h of infection or culture and observed using Wright Giemsa stain with light microscopy at a magnification of 1,000×.

*Y*. *pestis* KIM6+ exhibited high level tellurite resistance with an MIC of 0.31 mg/mL (Fig 2A) and a black colony phenotype typical for the *ter* operon mediated tellurite reduction when plated on LB agar containing sub-inhibitory concentrations of Na_2_TeO_3_ (Fig 2C). The KIM6+ also exhibited a filamentous cellular morphology when grown in media containing sub-inhibitory concentrations of Na_2_TeO_3_ and intracellularly in RAW264.7 cells (Fig 3A and 3G).

The partial-length Δ*terZAB* mutant retained high level tellurite resistance and tellurite reductive phenotype, but lost the filamentous cellular morphology response in the presence of sub-inhibitory Na_2_TeO_3_ and during macrophage parasitism (Figs 2, 3B and 3H). In contrast, the growth of the full-length Δ*terZABCDE* mutant was inhibited by Na_2_TeO_3_ at >0.01mg/mL, lost tellurite reductive phenotype, but like the Δ*terZAB* mutant, the Δ*terZABCDE* mutant did not assume a filamentous cellular morphology in the presence of 0.03mg/mL Na_2_TeO_3_ or during mouse macrophage parasitism (Figs 2, 3C and 3I). These results suggest that the *terZAB* segment of the operon is involved in the filamentous cellular morphology response, whereas the *terCDE* portion may be involved in the high level tellurite resistance and reduction.

### Complementation of the *Y*. *pestis ter* mutants

**Fig 4:**
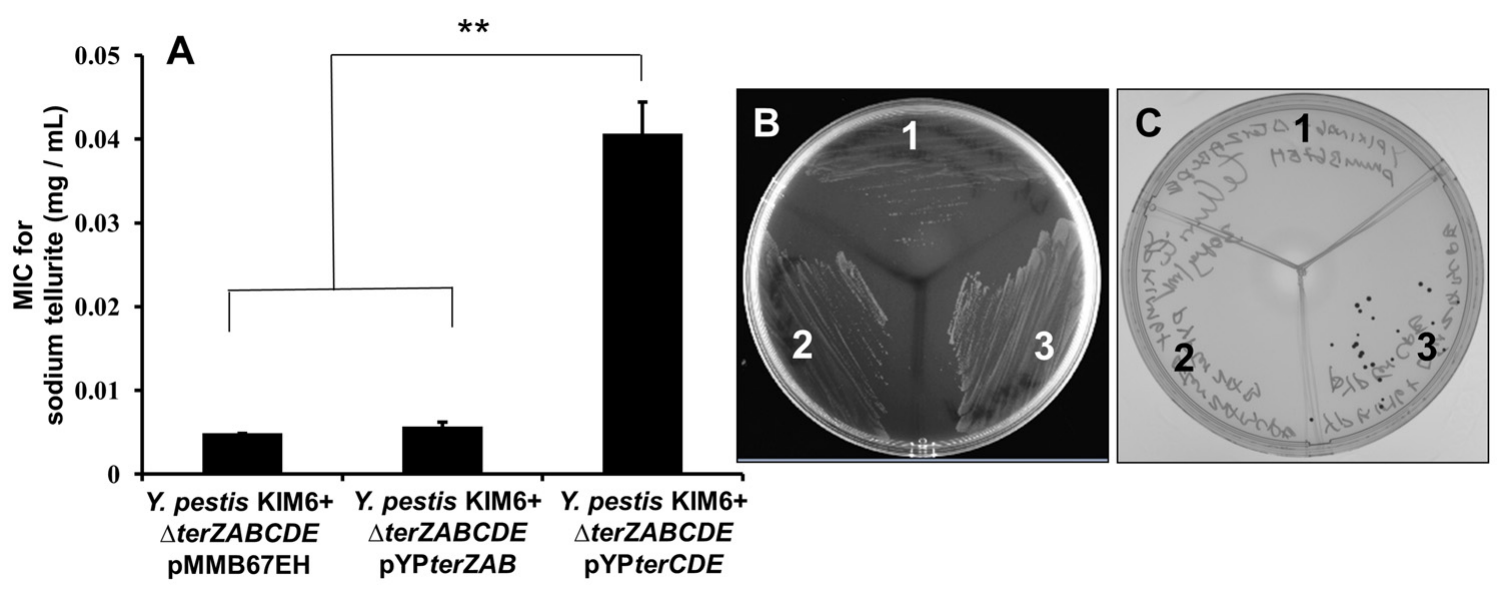
Minimal inhibitory concentration and reductive phenotype of*Y*. *pestis* KIM6+ *ΔterZABCDE* complemented with either *terZAB* or *terCDE* for Na_2_TeO_3_exposure. (A) *Y*. *pestis* KIM6+ *ΔterZABCDE* mutant transformed with an empty expression plasmid pMMB67EH or complemented with the expression plasmid carrying *Y*. *pestis terZAB* (pYP*terZAB*) *or terCDE* (pYP*terCDE*) genes were exposed to 2-fold serial dilutions of Na_2_TeO_3_ overnight at 28°C, and the MIC determined as the lowest tellurite concentration with no growth observed. Asterisk represents significant difference between the mean values (** denotes p<0.01). LB agar plates containing 0.1 mg/mL of Na_2_TeO_3_(C) or equivalent volume of PBS (B) inoculated with *Y*. *pestis* KIM6+ *ΔterZABCDE* mutant transformed with an empty expression plasmid pMMB67EH (1) or complemented with the expression plasmid carrying *Y*. *pestis terZAB* (pYP*terZAB*) (2)or *terCDE* (pYP*terCDE*) (3) genes and incubated at 28°C for 2 to 3 days. Black colony color was associated with tellurite reductive phenotype.

To further understand the roles of the various *ter* operon genes in tellurite resistance and the *Y*. *pestis* intracellular filamentous response, the full-length Δ*terZABCDE* mutant was complemented with either *terZAB* or *terCDE* in an expression plasmid with an IPTG inducible *tac* promoter. In the absence of the IPTG inducer, the *Y*. *pestis* Δ*terZABCDE* mutant complemented with *terZAB* did not restore tellurite resistance or tellurite reductive phenotype (Fig 4A and 4C), but complementation of the Δ*terZABCDE* mutant with *terCDE* in the absence of IPTG had low level resistance to tellurite with a MIC of 0.041 ± 0.013 mg/mL and restored the tellurite reductive phenotype (Fig 4A and 4C). Others have observed that the *tac* promoter allows low level protein expression even in the absence of IPTG inducer [26], which may account for partial restoration of tellurite resistance and reductive phenotype in the absence of inducer IPTG. In the presence of 0.025 mM IPTG, the MIC for tellurite for the Δ*terZABCDE* mutant complemented with *terCDE* increased to 0.073 ± 0.014 mg/mL, but the MIC for the Δ*terZABCDE* mutant complemented with *terZAB* remained at <0.01 mg/mL.

**Fig 5:**
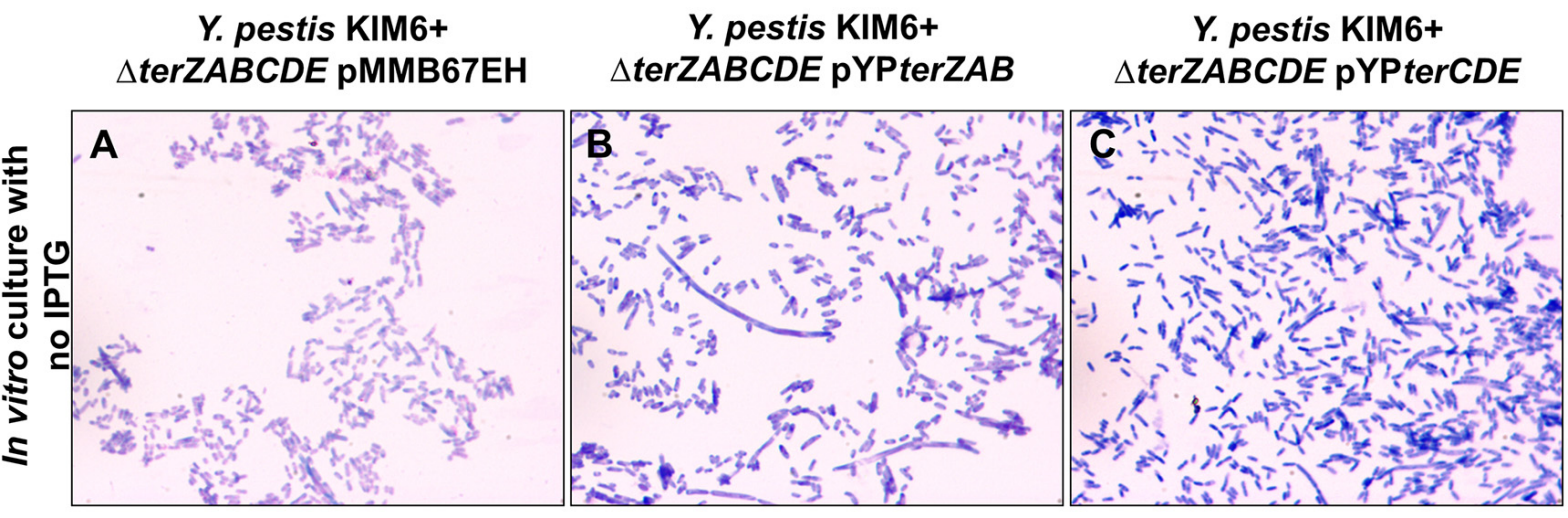
Cellular morphology of *Y*. *pestis* KIM6+ *ΔterZABCDE* complemented with either *terZAB* or *terCDE* in extracellular media. *Y*. *pestis* KIM6+ *ΔterZABCDE* mutant transformed with an empty expression plasmid pMMB67EH (A) or complemented with the expression plasmid carrying *Y*. *pestis terZAB* (pYP*terZAB*) (B) or *terCDE* (pYP*terCDE*) (C) genes were cultured in RPMI-1640 media without IPTG. Samples were collected at 2.5 h and observed using Wright Giemsa stain with light microscopy at a magnification of 1,000×.

**Fig 6:**
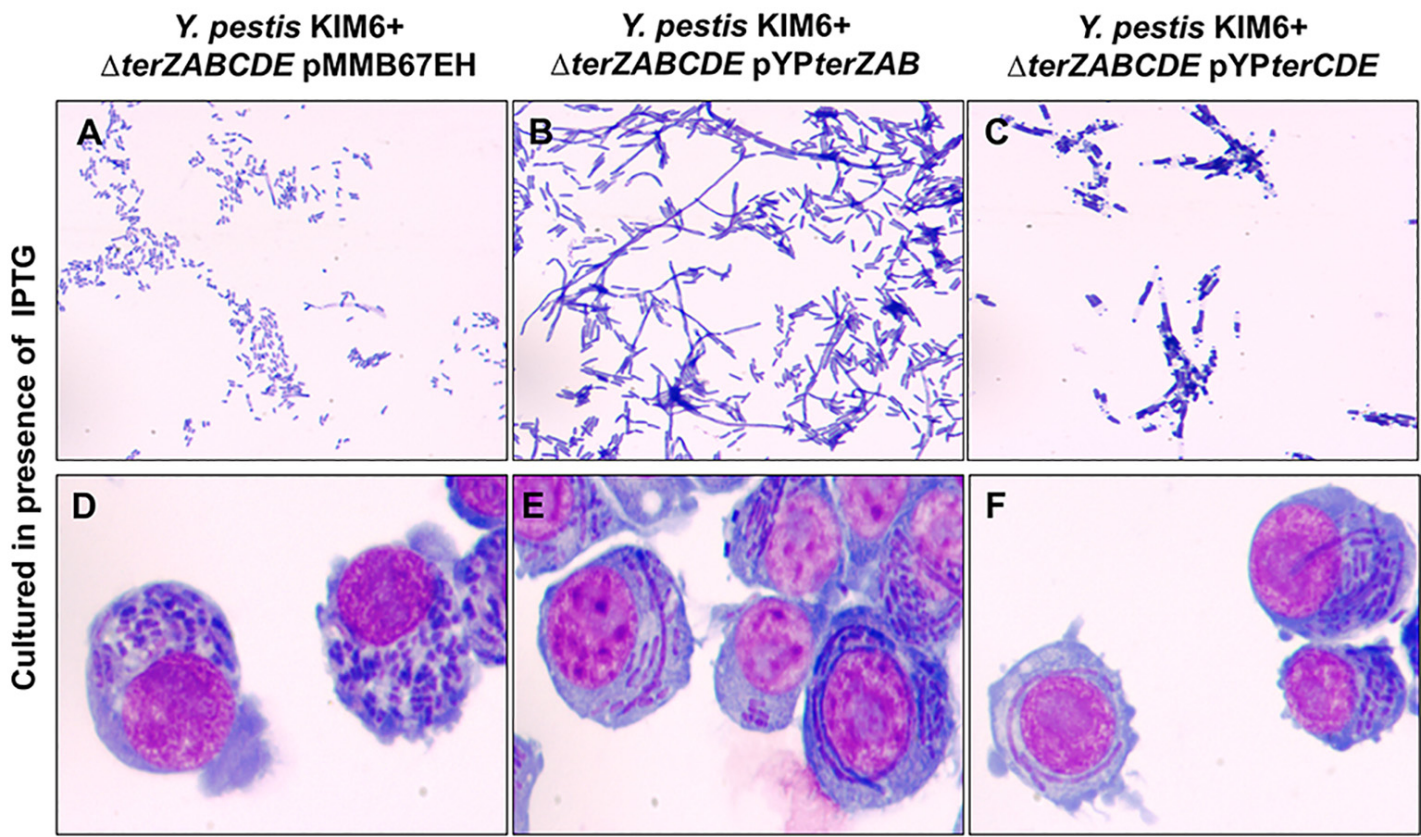
Cellular morphology of *Y*. *pestis* KIM6+ *ΔterZABCDE* complemented with either *terZAB* or *terCDE* in extracellular media or during intracellular parasitism. For panels A-C, *Y*. *pestis* KIM6+ *ΔterZABCDE* mutant transformed with an empty expression plasmid pMMB67EH (A) or complemented with the expression plasmid carrying *Y*. *pestis terZAB* (pYP*terZAB*) (B) or *terCDE* (pYP*terCDE*) (C) genes were cultured in RPMI-1640 media containing 1 mM IPTG for A and C, but 0.05 mM IPTG for B. For panels D-F, KIM6+ *ΔterZABCDE* mutant transformed with an empty expression plasmid pMMB67EH (D) or complemented with the expression plasmid carrying *Y*. *pestis terZAB* (pYP*terZAB*) (E) or *terCDE* (pYP*terCDE*) (F) genes infected RAW264.7 cells cultured in 0.05 mM IPTG. Sampled were collected at 2.5 h and observed using Wright Giemsa stain with light microscopy at a magnification of 1,000×.

The effect of *ter* complementation on the cellular morphology of the KIM6+ Δ*terZABCDE* mutant appears complex. In the absence of IPTG induction, the Δ*terZABCDE* mutant complemented with *terZAB* exhibited filamentous morphology and the Δ*terZABCDE* mutant complemented with *terCDE* exhibited some elongated rods (Fig 5B and 5C). In the presence of 1 mM IPTG inducer, expression of the *terZAB* and the *terCDE* in the full-length *Y*. *pestis* KIM6+ Δ*terZABCDE* mutant reduced growth at 24 h by 100% and ≈20%, respectively (S2 Fig). In the presence of 0.05 mM IPTG, the Δ*terZABCDE* mutant complemented with *terZAB* exhibited enhanced filamentous cellular morphology compared to its morphology in the absence of IPTG (Fig 6B versus Fig 5B). The Δ*terZABCDE* mutant complemented with *terCDE* in the presence of 1mM IPTG showed an altered cellular morphology including some elongated rod forms and altered nucleoid morphology (Fig 6C versus Fig 5C). However, both the Δ*terZABCDE* mutant complemented with *terZAB* or *terCDE* exhibited filamentous cellular morphology intracellularly in RAW 264.7 cells treated with 0.05 mM IPTG (Fig 6E and 6F).

### Expression of Y. pestis terZAB or terCDE in E. coli DH5α

**Fig 7:**
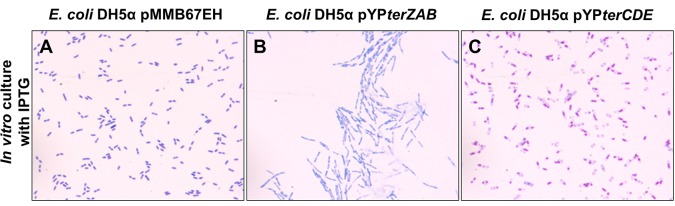
Cellular morphology of *E*. *coli* DH5α transformed with *Y*. *pestis terZAB* or *terCDE*. *E*. *coli* DH5α transformed with an empty expression plasmid pMMB67EH (A) or with the expression plasmid carrying *Y*. *pestis terZAB* (pYP*terZAB*) (B)or *terCDE* (pYP*terCDE*) (C) genes were grown in LB broth containing 1 mM IPTG. Samples were collected at 2.5 h of culture and observed using Wright Giemsa stain with light microscopy at a magnification of 1,000×.

To better understand the potential roles of *terZAB* versus *terCDE* in the filamentous morphologic response, *E*. *coli* DH5α which lacks a *ter* operon was transformed with the expression plasmids of *Y*. *pestis terZAB* or *terCDE*. Like the effect of higher level expression of the *terZAB* complement in *Y*. *pestis*Δ*terZABCDE* mutant, induction of *terZAB* complement by IPTG concentrations >0.1 mM resulted in decreased growth of the *E*. *coli* DH5α *terZAB* transformant but higher expression of the *E*. *coli* DH5α *terCDE* transformant induced by IPTG had no effect on growth (S2 Fig). These *E*. *coli* DH5α transformants did not exhibit tellurite resistance in the absence or presence of the IPTG inducer. The *E*. *coli* DH5α transformants also exhibited minimal cellular morphologic change in the absence of induction (supplemental 1% glucose and no IPTG). The *E*. *coli* DH5α transformant expressing *Y*. *pestis terZAB* induced by 1 mM IPTG exhibited filamentous cellular morphology, whereas the transformant expressing *terCDE* did not (Fig 7B and 7C). However, the *E*. *coli* DH5α *terCDE* transformant did appear to have altered, more condensed, nucleoid morphology (Fig 7C).

## Discussion

Several bacterial pathogens employ intracellular parasitism of host macrophages as a strategy to evade host innate immunity [27]. A common mechanism bacterial pathogens use to avoid macrophage killing is modification of the host phagosomal vesicle [28]. However, prior to this modification, phagocytized bacteria are subjected to stressful environments to which they must adapt to survive. A recently proposed survival strategy for intracellular bacteria is morphologic plasticity, and in particular, assumption of filamentous cellular morphology [12]. *Mycobacterium tuberculosis* and *Salmonella enterica* serovar Typhimurium have been observed to assume filamentous cellular morphology in response to phagocytosis by host macrophages; however, each pathogen appears to employ this adoptive response under different circumstances [29,30]. *S*. *enterica* serovar Typhimurium assumes a filamentous cellular morphology early during its intracellular parasitism of γ-INF activated mouse RAW246.7 macrophages in response to activation of macrophage MEK/ERK effectors and phagocyte NADPH oxidase activity [30]; whereas,*M*. *tuberculosis* assumes a filamentous morphology at longer times of intracellular parasitism possibly associated with transition to an alternative hypoxic-induced physiologic, non-replicative persistent state [29,31]. We recently reported that up to 7% of *Y*. *pestis* in mouse macrophages assumes a filamentous cellular morphology early in macrophage parasitism [15]. At later times in macrophage parasitism, *Y*. *pestis* modifies the YCV to spacious compartments and reassumes coccobacillary cellular morphology. Although no decisive experiment has been carried out to quantify the potential survival advantages of filamentous cellular morphology during intracellular parasitism, this morphologic change may help bacteria by 1) filaments causingphysical stress resulting in spacious enlargement of phagolysosome and dilution of antibacterial factors, 2) delaying cellular division reducing the transmission of the potentially damaged parental genomes to daughter cells until the SOS DNA repair system has had time to repair macrophage-induced reactive oxygen species damage to genomic DNA, and 3) diminishing re-phagocytosis of filamentous extracellular bacteria [9].

Others have observed filamentous growth of *Y*. *pestis* in experimentally infected guinea pig peritoneal macrophages and under *in vitro* culture conditions simulating the macrophage phagosomal environment of low Ca^+2^ or Mg^+2^[13,14,32]. Pujol and colleagues reported detection of filamentous *Y*. *pestis* in mouse bone marrow-derived macrophages; however, in contrast to what we observed, these filamentous forms were present only in a mutant for genes y2313-16 at 24 h in modified spacious YCV [33]. Assumption of filamentous cellular morphology by bacteria has been previously viewed as a non-specific response to chemical and physical conditions which slow bacterial growth [34], and not necessarily as a physiologically or pathogenically relevant survival mechanism. Therefore, what are the mechanisms involved in assumption of filamentous cellular morphology by *Y*. *pestis* and could these mechanisms be relevant to its pathogenesis?

Whelan and colleagues (1997) observed that cloning and expression of the R478 self-transmissible plasmid *ter* operon into *E*. *coli* DH5α resulted in adoption of filamentous cellular morphology for the transformed strain[25]. We observed that intracellular *Y*. *pestis* selectively expressed *ter* operon proteins TerD and TerE. Therefore, we hypothesized that the filamentous morphological response of intracellular *Y*. *pestis* in macrophages is mediated by proteins expressed from *ter* operon. We found that deletion mutation for *terZABCDE* resulted in loss of tellurite resistance and intracellular filamentous morphology elicited by sub-lethal concentrations of Na_2_TeO_3_ or intracellular parasitism. These findings support our speculation that the *ter* operon mediates the intracellular filamentous morphology of *Y*. *pestis* in macrophages. However, how the expression of Ter proteins leads to phenotypic change to the filamentous shape in *Y*. *pestis* is still an elusive concept. In addition to the proteins from *terZABCDE* operon, TehB, another Te^R^ determinant (*tehB*) from the *kil* locus of plasmid RK2 of *Streptococcus pneumonia*, causes the host or expression in a heterologous bacteria to assume filamentous shape when overexpressed under experimental conditions[35,36]. These results may suggest that the accumulation of proteins from the *ter* operon or from other Te^R^ determinants in the bacterial cells leads to activation or inactivation of a tellurite resistance dependent global, as yet uncharacterized, cellular mechanism by which the normal functions of the bacterial cell division machinery are interrupted.

Using transposon mutagenesis, Whelan and colleagues (1997) observed that the filamentous phenotype of *E*. *coli* DH5α was contained within the *terZABC* portion of the operon[25]. Similarly, we observed that deletion of *terZAB* resulted in loss of filamentous cellular morphology of *Y*. *pestis* exposed to sub-lethal concentrations of Na_2_TeO_3_ or intracellular parasitism, while deletion of the entire *ter* operon resulted in loss of filamentous response and tellurite resistance. Complementation of the *Y*. *pestis terZABCDE* deletion mutant with a plasmid carrying the *terZAB* restored the filamentous morphologic response to intracellular parasitism, but did not restore tellurite resistance; whereas complementation with *terCDE* partially restored the filamentous phenotype as well as the tellurite reductive phenotype. These phenotypes were observed in the absence of IPTG induction of expression by the *tac* promoter, suggesting that low level expression of the *ter* operon is sufficient to confer these phenotypes. In fact, when IPTG was used to gain higher expression levels of *terZAB*, this resulted in lethality.

Whelan and colleagues (1997) also noted that cloning *terZABCDEF* into *E*. *coli* DH5α in the absence of an upstream segment containing protective *terXYW* was lethal. Further investigation found that lethality resided in the *terZA* segment of the *ter* operon and that *terW* was protective for lethality [25,37]. In contrast to the toxicity of R478 *ter* operon in *E*. *coli* DH5α, expression of the *Y*. *pestis terZAB* or *terCDE* in *E*. *coli* DH5α was not lethal, even when expression was induced with IPTG, which instead elicited a filamentous phenotype in *E*. *coli* DH5α for the *terZAB* transformant. It would appear from our research reported herein and that of Whelan and colleagues, that *terZAB* genes are involved in the filamentous cellular morphologic response of *Y*. *pestis*. It is also likely that *Y*. *pestis* loci *terXYW* may be involved in bacterial filamentous morphological response as a regulatory element, but further studies are needed to characterize the role of each of these genes.

In our earlier proteomics study of *Y*. *pestis* during intracellular parasitism of mouse RAW264.7 macrophages, we reported expression of several general stress response proteins previously implicated in pathogenesis of *Y*. *pestis* or other intracellular bacterial pathogens as well as the novel expression of TerD and TerE proteins of the *Y*. *pestis* tellurite resistance operon *terZABCDE*[16]. We observed that the presence of TerD and TerE proteins in intracellular *Y*. *pestis* correlated with increase transcripts for *Y*. *pestis ter* genes during the first 2 hours of macrophage infections [38]. Furthermore, we observed increased expression of *ter* genes during exposure of *Y*. *pestis* to sub-lethal concentrations of Na_2_TeO_3_, implying two general possibilities. This chemical molecule may directly act on promoter region of *Y*. *pestis ter* operon sequence likely with the help of yet uncharacterized host factors, thus favoring enhanced expression of the operon. Alternatively, *ter* operon expression may be part of the global stress responses by *Y*. *pestis* to the exposure of Na_2_TeO_3_metal toxin similar to the responses observed in *Pseudomonas pseudoalcaligenes* [39]. In support of this, a recent bioinformatic analysis of the *ter* gene clusters using comparative genomics, sequence profiling, and structural analysis revealed that the *ter* clusters may “constitute a previously underappreciated, chemical stress and anti-viral defense system of bacteria”[40]. From this analysis, it has been suggested that TerBCD proteins may constitute a metal binding, sensing membrane complex which interacts with various proteins to produce nucleoside-like metabolites affecting DNA-processing. A study pertaining to protein-protein interactions reveals that Ter proteins can form homotypic and heterotypic interactions with other members of the Ter proteins. Protein TerD and TerE form strong heterodimeric complexes. Further, protein localization analyzes indicate that TerC may serve as a membrane protein and on its cytoplasmic side interacts with TerB and TerE, and the latter of these proteins further associates with TerD present in the cytoplasm [41]. These findings support the speculation that the Ter proteins are involved in as yet uncharacterized bacterial stress recognizing mechanism operating during infection processes.

## Materials and Methods

### Bacterial strains and growth conditions

*Y*. *pestis* and *E*. *coli* DH5α strains used for this study are listed in Table 1. Briefly, these strains were grown on either Brain Heart Infusion (BHI) (Difco, Becton Dickinson Company, Franklin Lakes, NJ) or Luria-Bertani (LB) (Sigma-Aldrich, St Louis, MO) agar plates and then isolated colonies were inoculated into either BHI broth and cultured overnight at 26°C with shaking at160 rpm for *Y*. *pestis* strains or LB broth and cultured overnight at 37°C with shaking at160 rpm for *E*. *c*oli and *Y*. *pestis* strains. Depending on selectable markers carried by various strains, 30 μg/mL of chloramphenicol (Sigma-Aldrich), 35 μg/mL of kanamycin (Sigma-Aldrich), 100 μg/mL of ampicillin (Sigma-Aldrich) or combination of these antibiotics was added to the bacterial growth media.

**Table 1:** Bacterial strains and plasmids used in this study.

Bacterial strains or plasmids		Genotypic characteristics	Source
*Y*. *pestis*			
	KIM62053.1+ (*Y*. *pestis* KIM6+)	Biovar Medievalis, *hms* ^+^ *psn* ^+^ *psa* ^-^ (Δ*psa*2053.1) *ybt* ^+^ *lcr* ^-^	[43]
	KIM6+ pKOBEG-*sacB*	*Y*. *pestis* KIM6+ was transformed with plasmid pKOBEG-*sacB*, Cm^R^	This study
	KIM6+ Δ*terZAB* pKOBEG-*sacB*	*Y*. *pestis* KIM6+ pKOBEG-*sacB* carries in-frame deletion mutation for genes *terZAB*, Cm^R^, Km^R^	This study
	KIM6+ Δ*terZAB*	pKOBEG-*sacB* plasmid cured *Y*. *pestis* KIM6+ Δ*terZAB* pKOBEG-*sacB*, Cm^S^, Km^R^	This study
	KIM6+ Δ*terZABCDE* pKOBEG-*sacB*	*Y*. *pestis* KIM6+ pKOBEG-*sacB* carries in-frame deletion mutation for genes *terZABCDE*, Cm^R^, Km^R^	This study
	KIM6+ Δ*terZABCDE*	pKOBEG-*sacB* plasmid cured *Y*. *pestis* KIM6+ Δ*terZABCDE* pKOBEG-*sacB*, Cm^S^, Km^R^	This study
	KIM6+ Δ*terZABCDE* pMMB67EH	*Y*. *pestis* KIM6+ Δ*terZABCDE* was transformed with plasmid pMMB67EH, Km^R^, Ap^R^	This study
	KIM6+ Δ*terZABCDE* pYP*terZAB*	Transformed with plasmid pYP*terZAB*, Km^R^, Ap^R^	This study
	KIM6+ Δ*terZABCDE* pYP*terCDE*	Transformed with plasmid pYP*terCDE*, Km^R^, Ap^R^	This study
*E*. *coli*			
	DH5α	F–Φ80*lac*ZΔM15 Δ(*lac*ZYA-*arg*F) U169 *rec*A1 *end*A1 *hsd*R17 (rK–, mK+) *pho*A *sup*E44 λ–*thi*-1 *gyr*A96 *rel*A1	Invitrogen^®^
	DH5α pMMB67EH	*E*. *coli* DH5α was transformed with plasmid pMMB67EH, Ap^R^	This study
	DH5α pYP*terZAB*	Transformed with plasmid pYP*terZAB*, Ap^R^	This study
	DH5α pYP*terCDE*	Transformed with plasmid pYP*terCDE*, Ap^R^	This study
Plasmids			
	pKOBEG-*sacB*	*λ*phage *redγβα* operon was cloned under the control of pBAD promoter. Gene *sacB* was also cloned in the back bone of this plasmid. Cm^R^	[42]
	pMMB67EH	Expression plasmid carries IPTG inducible *tac* promoter, Ap^R^	[44]
	pYP*terZAB*	Genes *terZAB* from *Y*. *pestis* KIM6+ were cloned into pMMB67EH between *Eco*RI and *Xba*I sites, Ap^R^	This study
	pYP*terCDE*	Genes *terCDE* were cloned into pMMB67EH between *Eco*RI and *Xba*I sites, Ap^R^	This study

### Tissue culture cells, growth conditions, infection procedures and light microscopic examinations

Mouse macrophage-like cell line RAW264.7 (ATCC, Manassas, VA) was used to assess intracellular parasitism by the various *Y*. *pestis* KIM6+ strains. The RAW264.7 cells were cultured in 15 mL of RPMI-1640 media (Sigma-Aldrich) containing 10% Fetal Bovine Serum (FBS) (Hyclone laboratories, Logan, UT) with or without with 100 μg/mL of ampicillin (as indicated) in 75 cm^2^ tissue culture flasks at 37°C with 5% CO_2_ tension.

RAW264.7 cells were infected with the various *Y*. *pestis* strains at a multiplicity of infection of five bacteria per RAW264.7 cell for 30 min at 37°C with 5% CO_2_ as described previously [15]. Subsequently, infected adherent cells contained in 75 cm^2^ tissue culture flasks were gently washed thrice with 15 mL of PBS and incubated for 2 h in 15 mL of RPMI media with 10% FBS supplemented with 50 μg/mL gentamicin (Sigma-Aldrich) in order to kill any residual extracellular *Y*. *pestis*. Following gentamicin treatment, the infected macrophages were once again washed thrice with 15 mL of PBS, released from the monolayer using a cell scrapper and finally fixed on microscopic slides by centrifugation at low speed for 5 min in a cytocentrifuge (Statspin Cytofuge, Norwood, MA). The slides were stained with Wright-Giemsa stain and examined using light microscope at 1,000× magnification.

### Genetic manipulations of *Y*. *pestis* strain KIM6+

The Lambda phage Red recombination system was used to construct the *Y*. *pestis* tellurite resistance operon mutants [42]. *Y*. *pestis* strain KIM6+ was grown in LB broth to ≈0.3 OD_600_ at 26°C with 220 rpm shaking. To prepare the culture for electroporation, bacteria were washed thrice with sterile water and collected by centrifugation at 1,000×*g* for 10 min at 4°C and adjusted to a 200-fold higher concentration in sterile water. Fifty microliters of this *Y*. *pestis* suspension was mixed with 200 ng of pKOBEG-*sacB* plasmid and subjected to electroporation at 1.8 KW for 5 ms using a MicroPulser electroporation apparatus (BioRad, Richmond, CA). The suspension was immediately transferred to LB broth containing an additional 1% D-glucose and incubated at 26°C for 2 h with 220 rpm shaking. One hundred microliters of this culture was subsequently streaked on LB agar plate containing 30 μg/mL chloramphenicol, and cm^r^ colonies (*Y*. *pestis* strain KIM6+ pKOBEG-*sacB*) were selected. This *Y*. *pestis* strain KIM6+ pKOBEG-*sacB* was grown to ≈0.2 OD_600_ in LB broth, and then to this culture, L-arabinose was added to the final concentration of 0.4% and incubated at 37°C for 4 h with 220 rpm shaking. Electrocompetent *Y*. *pestis* strain KIM6+ pKOBEG-*sacB* was prepared as described above for KIM6+.

Polymerase chain reaction was used to construct a DNA fragment coding for a constitutively expressed kanamycin resistance gene (B-Bridges International, Cupertino, CA) flanked by 50 nucleotides homologous to up and downstream sequences of gene deletion region of the *ter* operon in *Y*. *pestis* chromosome. One of the fragments was designed to delete *Y*. *pestis* strain KIM6+ *ter* operon genes *terZAB* and another for genes *terZABCDE*. Each of these fragments was individually electroporated into *Y*. *pestis* strain KIM6+ pKOBEG-*sacB* using a final concentration of 750 ng in 50 μL at 1.8 KW for 5 ms. The electroporated bacterial suspension was immediately transferred to LB broth supplemented with 1% D-glucose and incubated at 37°C for 3 h with 220 rpm shaking. Subsequently, 100 μL of this culture was streaked on chloramphenicol and kanamycin LB agar plates, and the resulting Cm^R^ and Km^R^ colonies were assessed for recombination at the intended site on *Y*. *pestis* chromosome using PCR. The pKOBEG-*sacB* plasmid was cured from the *Y*. *pestis* strain KIM6+ pKOBEG-*sacB* Δ*terZAB* or Δ*terZABCDE* strains by plating on kanamycin LB agar plate containing 10% sucrose.

### Determination of minimum inhibitory concentration (MIC) of Na_2_TeO_3_for *Y*. *pestis* and *E*. *coli* DH5α strains

*Y*. *pestis*KIM6+, *Y*. *pestis* KIM6+ Δ*terZAB* and *Y*. *pestis* KIM6+Δ*terZABCDE* grown in BHI broth as describe above were diluted in LB broth to prepare the working inocula of 2×10^6^ CFU/mL. For MIC determinations, 50 μL of 10 mg/mL Na_2_TeO_3_ in LB broth was two-fold serially diluted in wells A1-G1 through A11-G11 containing 50 μL of LB broth yielding a Na_2_TeO_3_concentration gradient of 5 to 0.00488 mg/mL of Na_2_TeO_3_. Wells in column H1-12 contained 50 μL LB broth as a control. Wells A1-H12 were inoculated with 50 μL/well of the above working inoculum to yield a final inoculum level of 1×10^6^ CFU/mL. The plate was incubated at 28°C for overnight, and the minimum concentration of Na_2_TeO_3_ at which no visible growth of the bacteria observed was recorded as MIC.

For MIC determinations using *Y*. *pestis* KIM6+ Δ*terZABCDE* and *E*. *coli* strain DH5α complemented with pYP*terZAB* or pYP*terCDE*, addition of 25 μL/well of LB broth contained 0.1 mM Isopropyl β-D-1-thiogalactopyranoside (IPTG) (Promega, Madison, WI) and 0.4 mg of ampicillin were added to each well to yield a final concentrations of 25 μM IPTG and 100 μg/mL ampicillin, and a 25 μL/well inocula of a 4×10^6^ CFU/mL working inocula was used to infect wells.

### Tellurite reductive phenotype of *Y*. *pestis* strains

To assess the tellurite reductive phenotype, *Y*. *pestis* KIM6+ and the *terZAB* and *terZABCDE* mutants and the *terZABCDE* mutant complemented with pYP*terZAB* or pYP*terCDE* were streaked on LB agar plate containing 30 μg/mL of Na_2_TeO_3_or an equivalent volume of PBS. These plates were incubated at 26°C for 48 to 72 h, and the reductive tellurite phenotype assessed by the accumulation of metallic Te^0^assessed visualized by the presence of black colonies [18].

### Light microscopic examination of *Y*. *pestis* strains treated with Na_2_TeO_3_

*Y*. *pestis* KIM6+strains were cultured in RPMI-1640 media with 10% FBS containing 30 μg/mL Na_2_TeO_3_ or equivalent volume sterile PBS for 2.5 h at 26°C with 160 rpm shaking. Subsequently, samples from each of these cultures were spun onto the microscopic slides at moderate speed for 5 min using a cytocentrifuge. The slides were stained with Wright Giemsa stain and examined via light microscopy at 1,000× magnification.

### Light microscopic examination of *Y*. *pestis* and *E*. *coli* DH5α strains grown in glucose or IPTG supplemented media

*Y*. *pestis* KIM6+ and *E*. *coli* DH5α strains transformed with plasmids carrying *terZAB* or *terCDE* genes were examined under light microscope to evaluate the morphological changes caused by expression of these genes. Overnight cultures of *Y*. *pestis* KIM6+ or *E*. *coli* DH5α strains were diluted to 1×10^6^ CFU/mL in LB broth containing 1% extra D-glucose (Sigma-Aldrich) or 0.05 or 1 mM IPTG and 100 μg/mL ampicillin as the final concentrations. These cultures were incubated at 26°C for *Y*. *pestis* strains and at 37°C for *E*. *coli* DH5α strains for 5 h with 160 rpm shaking. Subsequently, the samples were spun on glass slides using cytocentrifuge, stained with Wright Giemsa stain and observed under light microscope at 1,000× magnification

### Complementation of tellurite resistance genes in*Y*. *pestis* strain KIM6+ Δ*terZABCDE*mutants

Gene clusters *terZAB* or *terCDE* from *Y*. *pestis* strain KIMK6+ were cloned in an expression plasmid pMMB67EH under IPTG inducible ‘*tac*’ promoter and then transformed to *Y*. *pestis* strain KIM6+ Δ*terZABCDE*. The open reading frames (ORFs) and intergenic non-coding sequences for gene clusters *terZAB* and *terCDE* were amplified from *Y*. *pestis* strain KIM6+ genome by using primers in Table 2 and the Phusion^®^ High-Fidelity PCR Kit (New England Biolabs). The resulting PCR products were purified using Wizard^®^ SV gel and PCR clean-up system (Promega), and then 2 μg each of the fragments were digested with *Eco*RI-HF^™^ and *Xba*I (New England Biolabs) in 100 μL according to the manufacturer’s instruction. Subsequently, the digested PCR products were once again purified and used for the downstream applications.

**Table 2:** Oligonucleotides used in this study.

**Oligonucleotides**	**Sequence Information**	**Source**
**Δ*terZAB* or Δ*terZABCDE*-F / Δ*terZAB*-R**	5’-ATATGATTGGTGTGATGCGGTTAATGTGACTTGTTCAATAATTTCATTCTAATTAACCCTCACTAAAGGGCG-3’/ 5’-TTACAGTTGAAAATCAGCAGGGACAAAACCTAACGTCACGCAAATTTCACTAATACGACTCACTATAGGGCTC-3’	This study
**Δ*terZABCDE*-R**	5’-GCAGCAGGGTTTAATTGAAACGGGCAGTCGTTATACCGACACACCATGCTTAATACGACTCACTATAGGGCTC-3’	This study
**5’-*terZ*-F / 3’-*terE*-R**	5’–CAGTGGATGAATCCTTATGAAACCG-3’ / 5’–TCTTCTATCCGCCGAGTATACCC-3’	This study
**Kan-F /** Kan**-R**	5’–TATCAGGACATAGCGTTGGCTACC-3’ / 5’–CGAGACTAGTGAGACGTGCTAC-3’	Gene Bridges^®^
*terZ*-*Eco* **RI-F / *terB*-*Xba*I-R**	5’–GGGGGGGAATTCTTGTTCAATAATTTCATTCTAAA-3’/5’–GGGGGGTCTAGATTACAGTTGAAAATCAGCAG-3’	This study
*terC*-*Eco* **RI-F / *terE*-*Xba*I-R**	5’–GGGGGGGAATTCATGCACCGCCATGATAAGCC-3’/5’–GGGGGGTCTAGATTATACCGACACACCATGCT-3’	This study

The low copy number, prokaryotic expression plasmid pMMB67EH was isolated from *E*. *coli* strain DH5α harboring this plasmid using PureLink™ HiPure Plasmid Filter Midiprep Kit (Life Technologies, Grand Island, NY). Two microgram of this plasmid was digested with *Eco*RI-HF^™^ and *Xba*I in 100 μL volume, and then the digest was purified through PCR clean-up system (Promega). Finally, each one of the above purified PCR products was mixed individually with the digested plasmid at equal molar concentrations, and the cut ends were ligated by T4 DNA ligase (New England Biolabs) at 16°C for 30 min. This ligation mixture was used to transform *E*. *coli* strain DH5α by electroporation at 1.8 KW for 5 ms, and the resulting colonies were plated on ampicillin LB agar plates to select for insertion of PCR product into the plasmid. Later, the plasmid carrying desired DNA fragment confirmed by PCR was isolated from *E*. *coli* strain DH5α, and 500 ng of the plasmid was transformed into *Y*. *pestis* strain KIM6+ Δ*terZABCDE* through electroporation as described previously. Successful transformants were selected on LB agar plate containing 35 μg/mL kanamycin and 100 μg/mL ampicillin.

### Infection of RAW264.7 cell with *Y*. *pestis* KIM6+ Δ*terZABCDE* complemented gene clusters *terZAB* or *terCDE*

RAW264.7 cells were infected with *Y*. *pestis* KIM6+ Δ*terZABCDE* having complementation plasmids as described above, but with the following modifications. RAW264.7 cells were cultured overnight in RPMI media with 10% FBS supplemented 100 μg/mL ampicillin. Subsequently, throughout the infection period starting from 30 min bacterial exposure and the 2 h gentamicin treatment, RPMI media with 10% FBS containing 100 μg/mL ampicillin was used. In addition, during gentamicin treatment, IPTG was added to the culture media at the final concentration of 0.05 or 1mM, as indicated, and incubated for 2 h along with the antibiotics.

### Statistical analysis

For MIC data, the mean values from different experiments were analyzed using a one-way ANOVA followed by Tukey’s HSD Post-hoc test. The resulting significant differences were reported at p-value <0.01 or < 0.05.

## Supporting Information

S1 Fig Growth curves for *Y*. *pestis* KIM6+,*ΔterZAB* and *ΔterZABCDE* mutants. Isolated colonies from overnight growth of *Y*. *pestis* KIM6+,*ΔterZAB* and *ΔterZABCDE* mutants on BHI agar at 26°Cwere inoculated into triplet BHI broth cultures and cultured at (A) 26°C or (B) 37°C with shaking at160 rpm. Growth was assessed by OD_600nm_ reading. Error bars represent standard deviation from the mean.
(TIF)

S2 Fig Effect of IPTG induction of *ΔterZAB* and *ΔterZABCDE* complement expression on growth of *Y*. *pestis* KIM6+ *ΔterZABCDE* and *E*. *coli* DH5α. Isolated colonies from overnight growth of *Y*. *pestis* KIM6+ *ΔterZABCDE* mutant on BHI agar at 26°Cand *E*. *coli* DH5αon LB agar at 37°C were inoculated into triplet BHI broth for *Y*. *pestis* or LB broth for *E*. *coli* and cultured at 37°C with shaking at160 rpm for 24 h. Growth was assessed by OD_600nm_ readings and expressed as a fold change in OD_600nm_ from the 0 to 24 h. Error bars represent standard deviation from the mean.
(TIF)
