# Supplementary figures and images for: Role of Tellurite Resistance Operon in Filamentous Growth of Yersinia pestis in Macrophages
Source: PLoS One. 2015 Nov 4;10(11):e0141984. doi: 10.1371/journal.pone.0141984 (PMC4633105; doi:10.1371/journal.pone.0141984)

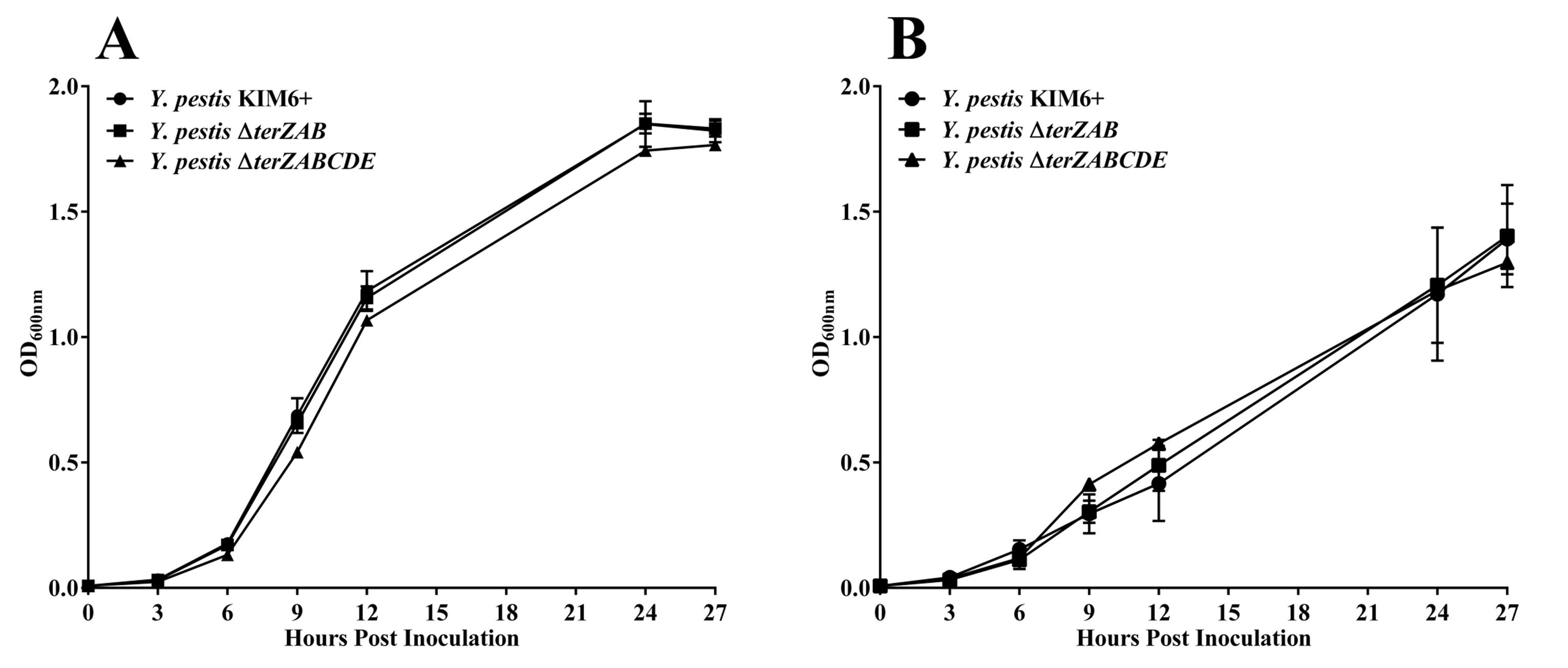

Supplement: S1 Fig — Isolated colonies from overnight growth of Y. pestis KIM6+,ΔterZAB and ΔterZABCDE mutants on BHI agar at 26°Cwere inoculated into triplet BHI broth cultures and cultured at (A) 26°C or (B) 37°C with shaking at160 rpm. Growth was assessed by OD600nm reading. Error bars represent standard deviation from the mean. (TIF) [file pone.0141984.s001.tif]

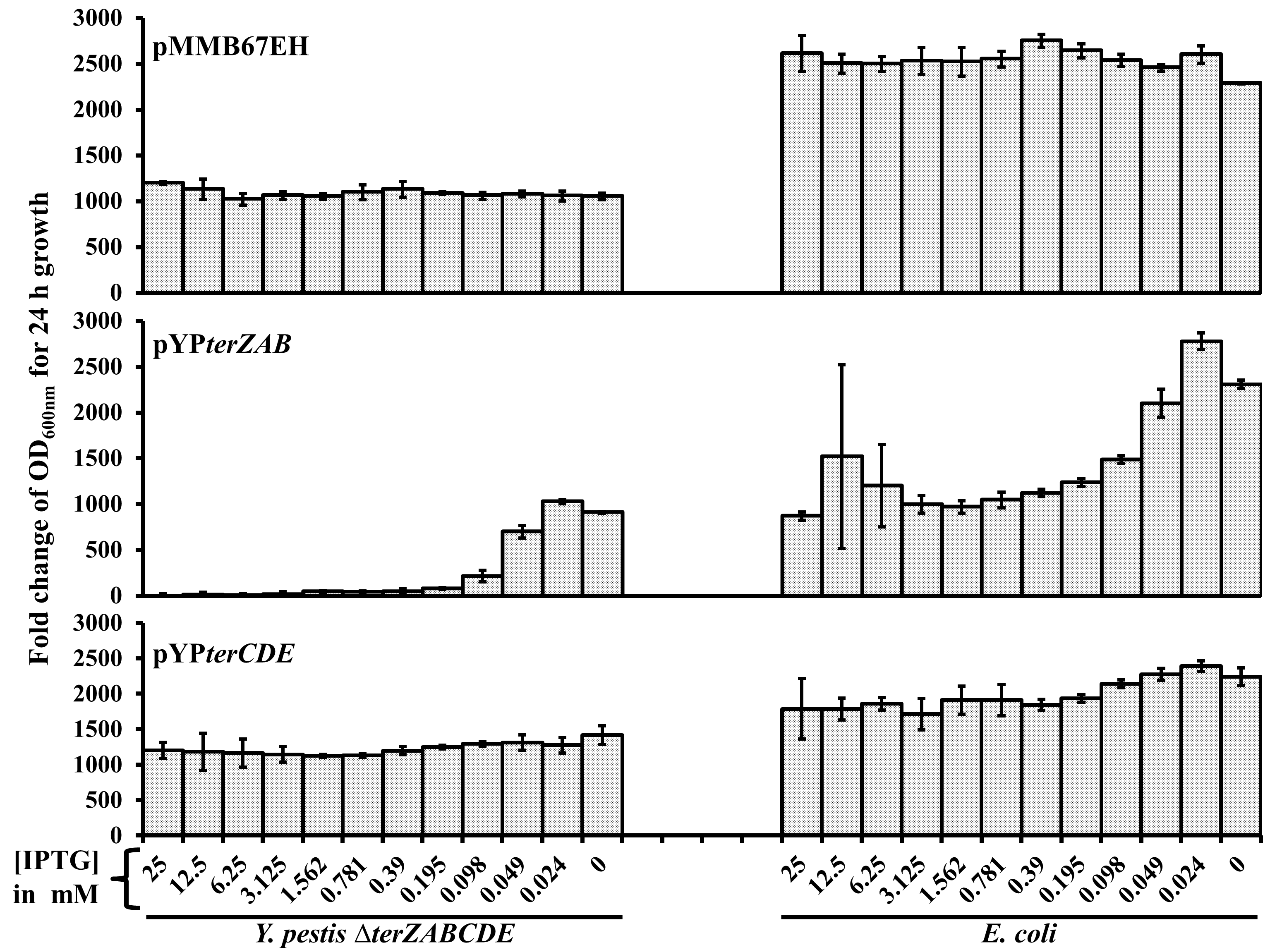

Supplement: S2 Fig — Isolated colonies from overnight growth of Y. pestis KIM6+ ΔterZABCDE mutant on BHI agar at 26°Cand E. coli DH5αon LB agar at 37°C were inoculated into triplet BHI broth for Y. pestis or LB broth for E. coli and cultured at 37°C with shaking at160 rpm for 24 h. Growth was assessed by OD600nm readings and expressed as a fold change in OD600nm from the 0 to 24 h. Error bars represent standard deviation from the mean. (TIF) [file pone.0141984.s002.tif]
